# Medical Extended Reality Trials: Building Robust Comparators, Controls, and Sham

**DOI:** 10.2196/45821

**Published:** 2023-11-22

**Authors:** Susan Persky, Luana Colloca

**Affiliations:** 1 Social and Behavioral Research Branch National Human Genome Research Institute Bethesda, MD United States; 2 Department of Pain and Translational Symptom Science, School of Nursing University of Maryland Baltimore Baltimore, MD United States

**Keywords:** augmented reality, clinical trial design, control conditions, medical extended reality, sham VR, virtual reality

## Abstract

The explosive pace of development and research in medical extended reality (MXR) is a testament to its promise for health care and medicine. In comparison with this growth, there is a relative sparsity of rigorous clinical trials that establish the efficacy and effectiveness of these interventions. Explicating mechanisms of action across clinical areas and MXR applications is another major area of need. A primary impediment to these goals is a lack of frameworks for trial design, more specifically, the selection of appropriate controls that effectively address unique elements of MXR. This paper delineates a framework for designing controls, sham conditions, and comparators, as well as proposed considerations for MXR trial designs. Special consideration is given to the design of sham conditions. Improved designs would enable more robust findings and the development of generalizable knowledge that could be adopted across MXR interventions.

## Introduction

### Overview

Medical extended reality (MXR), the application of extended reality (XR) technology such as virtual reality (VR) and augmented reality to health care and medicine, spans a variety of domains, including patient treatment, procedure support, health education, and provider training. MXR is an area of long-anticipated promise that is finally beginning to materialize and make its way into clinical implementation [1-6]. Accordingly, the pace of development and research in MXR has accelerated dramatically in the past few years, resulting in a flood of new products and scientific papers. By comparison, there is yet a sparsity of rigorous clinical trials that establish the efficacy and effectiveness of these interventions. There is also a major need for research that establishes mechanisms of action across clinical areas and application types. This work will allow for better targeting and tailoring of future intervention designs [7,8]. A primary impediment to these goals is the lack of frameworks for trial designs and the selection of appropriate controls that effectively address the unique elements of MXR. This paper posits the challenges inherent in clinical trial designs for MXR and proposes approaches for choosing controls, sham conditions, and comparators that account for the technology’s complexity and multifaceted nature.

### General Considerations

The clinical trial literature discusses comparators, an umbrella term describing another treatment, product, or experience to which the focal intervention is compared as a frame of reference to determine its efficacy, safety, or other benchmarks. Comparators as a class encompass the more specific concept of a control condition. A control condition provides a comparison that includes no active components of the intervention (ie, elements that underlie effectiveness) and, as such, allows researchers to determine that the treatment or intervention itself causally influenced patient outcomes. A control condition, therefore, includes elements present in the intervention that differ from the proposed primary active components (eg, intervention delivery vehicle and interactions with the study team), allowing researchers to rule out alternative explanations for patient outcomes. This may take the form of a sham (where study activities are performed without the performance of the active component activities) or a placebo (where an inactive component is substituted for an active one). In the context of MXR trials, there is little practical difference between sham and placebo control, and we use them interchangeably here.

The control condition essentially defines the claims that can be made about the efficacy or effectiveness of an MXR intervention in the case of a positive finding (ie, finding that the intervention is more effective than the control). As such, this is the primary consideration underlying control condition design or comparator choice. Due to variation in trial goals and variation in the nature of individual MXR interventions, there is no universal, optimal control for MXR interventions as a class. Control suitability depends on features of the intervention, the setting, and the target population and, ultimately, on potential sources of patient and health care provider expectancies (ie, beliefs about whether and how well a treatment will work).

There are many other factors that influence choice of comparator or control, and these should be considered in light of the trial’s primary objective. For example, different considerations are typically applied when designing control conditions for proof-of-concept, feasibility, and acceptability studies. There are also situations in which the profile of the intervention, the health condition under study, or the patient population dictates what is possible and ethically acceptable [9,10]. For example, it may be unethical to assign patients in ill-health to a no-treatment control when treatments are otherwise available. In addition to many potential benefits, there are several potential risks associated with MXR use that must be considered in the process of trial design. These include cybersickness (similar to motion sickness, stemming from XR use); strain or discomfort to the head, neck, or face; and distraction or disorientation [11]. Some approaches to MXR provision could also introduce risks to patient privacy if adequate data collection and protection measures are lacking or not possible in a given situation [12]. The risks present in the specific study and intervention context must be weighed against potential benefits for the ethical design of MXR trials.

Given competing demands, choosing the comparator or designing the control condition can be one of the most difficult, yet vitally important, parts of designing an MXR study, as it dictates what can be concluded from the study in terms of outcomes such as efficacy, effectiveness, acceptability, safety, and mechanisms of action.

### Existing Guidance

Given the unique profile of MXR, the research community has recognized the need for specialized study design. Birckhead et al [13] made recommendations for clinical trial design, covering multiple design questions, including choice of control group. The authors discuss a range of forms that a control condition can take inside or outside of a headset, including both passive and interactive features. Ultimately, they recommend a study design that considers purported MXR mechanisms and targets of action. Beams et al [14] similarly address a variety of challenges associated with the evaluation of MXR applications from a regulatory perspective. Among other possibilities, these authors describe the promise of sham control interventions that involve the provision of an XR headset that administers 2D visualizations. Such sham groups (discussed in detail below) have become popular in VR-based clinical trials, including the high-profile trials associated with early US Food and Drug Administration–cleared XR applications [14]. More broadly, scientists and regulators have noted that the subject of clinical trial design, and control conditions for MXR trials, needs more exploration and explication in the scientific literature [6,13-15]. Such consideration would greatly strengthen the quality of trials evaluating MXR treatments and interventions. Herein we focus on frameworks and control conditions for MXR generally, although many details and examples are based on VR given its current dominance of the MXR literature.

### Frameworks for MXR Trials

Although there are many variations in frameworks, the broad classes of randomized controlled trial comparators can be boiled down to those that are created and administered by the research team (eg, a control condition developed to parallel the intervention) and those that are not created by the research team (eg, treatment as usual, wherein participants are asked to continue their existing treatment approach). Gold et al [16] further break these areas down into several specific examples in the domain of traditional clinical trials. These include placebo control, specific factor component control, active comparator, treatment as usual, no treatment control, and waitlist control. We expand on these examples in Table 1 to consider how they can be applied to trials evaluating MXR interventions.

**Table 1:** Comparator group types, associated claims, and considerations.

Categories and definitions	MXR^a^ example	Example claim from a positive randomized controlled trial	Consideration for use in MXR trials
*Placebo control*: nonspecific elements of the intervention, such as attention or delivery mechanism, without the proposed active elements	Sham virtual reality	The MXR intervention is effective, and this is due to the purported active components	There is a clear set of proposed active components that can be separated from inactive elements Placebo has been tested, and its effects are understood Placebo can be made to appear credible to participants
*Specific factors component control*: reduced number of active intervention factors (ie, components) in addition to inactive factors	Intervention content delivered in 2D on a tablet	Active components of the intervention benefit from XR^b^ delivery	XR features are likely to boost or underlie effectiveness Some active components (eg, patient education content) can be disentangled from XR delivery
*Active comparator*: a different, evidence-based treatment	In-person physical therapy, provided by study	XR intervention works better than another specific available treatment	Quality alternatives are available Looking for equivalence (XR is “as good as”) Existing treatment is variable, or there are other benefits of standardizing the comparator
*Treatment as usual*: continued treatment as is typical for the health condition under study	Instructions to continue current therapy with the personal health care provider	XR intervention works better than typical available treatment in general	Alternatives are available Existing treatments are relatively standardized, or variance can be reduced or accounted for
*No treatment control*: no intervention elements provided	Providing nothing	XR intervention works better than doing nothing	There are no alternatives available Low potential for placebo effects
*Waitlist control*: no intervention, but with expectation of future treatment	Provide nothing but promise XR intervention later	XR intervention works better than doing nothing	There are no alternatives available Potential for nocebo (ie, negative expectations for outcomes) if not receiving treatment
*No control*: treatment group not compared with another group	A single group is assessed before and after use of XR intervention	Outcomes reported to be different after using XR than they were before	Inability to run control group Generally, this method is not acceptable for establishing causal relationships

^a^MXR: medical extended reality

^b^XR: extended reality

### Special Considerations for MXR Trials

Because a central question in MXR trial designs often involves determining whether and to what extent the XR delivery mechanism is an active element of the intervention, it is crucial to examine what is unique about XR in its contribution to interventions and treatments. MXR technologies combine aspects of other intervention platforms that are not typically co-occurring (eg, experiencing digital content through behaviorally driven action) and present new elements, such as the potential to embody a user in a separate digital body. Several frameworks present unique features of XR for various use cases that may be instructive [3,17-20]. At their core, these unique features are the elements of MXR that could either elicit significant placebo effects (eg, novelty and modulated input from closed-loop systems) or be an active component through which applications in XR achieve effectiveness (eg, experienced presence in a simulated environment and behaviorally driven interactivity), as depicted in Table 2.

**Table 2:** Selected features of extended reality (XR) and their implications for evaluating interventions.

XR features	Description	Implications for MXR^a^ interventions	Example approaches for control conditions
Immersion	Use of XR equipment can, to various degrees, surround the user or limit access to the physical surroundings and allow sensory access only to digital content	Direct influence on distraction and attention due to augmented or reduced access to the physical environment Blocking of other contextual stimuli orients attention and reduces distractions	Reducing outside sensory input such as using an XR headset for control stimuli Providing control stimuli in a darkened or bare (physical) room
Presence	XR engenders a sense of “being” in the virtual digital environment such that users feel they are existing within the virtual world or along with its elements	Presence is considered the hallmark of virtual reality Makes possible bodily illusions and experience of having personally engaged in a digital simulation, can underlie motivation and psychological influence, and can engender responses similar to real-world settings	Narrative approaches outside XR that cause presence and mental transportation Use of nontherapeutic XR applications that are similarly presence-inducing Use of the XR intervention environment with therapeutic elements removed or replaced
Embodiment	The ability for XR to make users feel as if they are existing within a digital body other than their own (eg, an avatar), enacting behaviors in the virtual environment	Ability to change perception and viewpoint and have experiences that are depersonalized or repersonalized in another body or entity Central to therapy related to absent body parts (eg, phantom limb syndrome) and some empathy-inducing experiences	Body transfer to a digital body or entity without the therapeutic element present Transfer to a digital body that engages in nontherapeutic activity Directed body movements or scripted experiences outside of the XR environment
Multisensory stimulation	XR can engage multiple senses such as visual (3D and stereoscopic), aural, olfactory, haptic, as well as senses not typically engaged by media (eg, proprioception)	Ability to present media interventions through more sensory channels than typical and engage more brain areas	Engage key sensory channels in the physical environment (eg, providing ambient scent or sound) Provide sensory content (eg, music) inside of XR that differs from active content but is similar on key factors (eg, salience and valence)
Behavior-driven	Real-time physical behaviors in XR environments have direct implications for experience and drive action	Allows multiple modes of interaction with whole-body input and facilitates an ability to engage in naturalistic behavioral activity Allows for fine-grained, continuous behavioral measurement	Engage physical movement and behavior outside of the XR environment (eg, guided movements or screen-based demonstration) Behavior in XR can drive nontherapeutic elements of experience (eg, moving hands to draw a picture rather than perform an exercise)
Interactivity	Response of XR environments to user input including physical movement and input from hand controllers	Integrates users directly into the action in a naturalistic way, increases engagement, and enables personalization	Interactive applications on non-XR platforms Interactive elements can trigger nontherapeutic elements such as trivia questions rather than patient education
Closed-loop systems	Behavior, performance, physiology, or other metrics drive stimuli provided by XR intervention	Ability to adjust intervention to user behavior or performance in real time for adaptive learning or training and provision of biofeedback	Provide noncontingent or faux feedback to the participant on performance Provide contingent feedback on a nontherapeutic factor (eg, based on EMG^b^ when intervention uses EEG^c^)
Novelty	Consumer adoption is growing, and familiarity is variable Expected to change over time with increased adoption There is often an intangible “wow” effect, especially for newer users	Wide-ranging attitudes toward application of the technology from skepticism, given entertainment applications, to increased expectations for efficacy and excitement Possibility for disconnect between user expectation and MXR intervention capabilities	Integrate aspects of the novel technology without specific features (eg, sham) Use other novel, emerging digital technology approaches (eg, wearables and artificial intelligence–based elements)
Form factor	The primary form factor is head mounted, although not exclusive	Can cause discomfort to the head and neck, eye strain, and heat retention, especially with extended use	Integrate a head-worn element with or without XR capabilities

^a^MXR: medical extended reality.

^b^EMG: electromyography.

^c^EEG: electroencephalography.

The role of XR elements in patient outcomes is likely to vary substantially between interventions. The goal for trial design, then, is to include elements that are likely to be confounding or elicit placebo effects while avoiding those that underlie the efficacy of active components. This last piece is critical because the closer a control condition matches the active elements of an intervention, the more difficult it may be to find an effect [21]. Arriving at a control that will ensure validity and support claims about an MXR intervention while also avoiding the suspected active components of the trial can be difficult, and trade-offs may be necessary. For example, a trial assessing whether a new XR intervention for surgical education is better than the leading computer-based approach will typically include the standard version of the computer-based approach as an active comparator and thus will not control for behaviorally driven XR interaction even though it is a purported active element of the intervention.

It is also crucial to note that XR itself, in terms of the equipment and general software approaches, is never “the intervention.” Rather it is the delivery vehicle for the content comprising the intervention, and it may or may not enable unique intervention features. Content can range widely across MXR interventions, from relaxing 360° nature videos to interactive experiences with artificial intelligence–based virtual humans and therapeutic video games, each of which requires different considerations. While this is not yet discussed explicitly in clinical research, XR design models in education specify the need to consider the interaction between XR hardware approaches and specific media content to support and optimize outcomes [20]. The content of the intervention, the chosen control condition, and the alignment between the 2 must be central considerations underlying decision-making.

Although each individual XR-relevant factor can be addressed by feature choices in a control condition, the reality is that features typically co-occur within a given intervention and must be considered in conjunction with one another. Features become important for interventions depending on their content. For example, in the case of relaxation and distraction-based pain management, features such as presence and multimodal sensory elements may be paramount active components for the intervention’s effect, whereas in a physical, gamified rehabilitation application, the behaviorally driven nature of XR and the ability to create a closed-loop system may be central. The novelty and form factor associated with the equipment could be useful to control across both interventions. Certainly, there are also many elements that are not specific to MXR that should also be considered for inclusion in controls, such as staff attention and interaction, training content, and educational content. The standardization of general intervention features such as these should not be overlooked in MXR interventions.

### Sham Control Design and Testing

While there are clearly a host of potential control condition designs, the sham control approach is worth deeper examination due to its popularity and promise. The term “sham” in the XR context has been used to describe a variety of approaches, including headsets with the power off, VR-based simulations of the physical research environment, and 2D content viewed outside of a headset [22-27]. Consensus in defining sham versus control in MXR is needed. Most often, a sham VR condition refers to watching a 2D video on a virtual screen in an XR headset, similar to watching a large screen television in the physical world. The headset provides a stereoscopic view and may track head rotation or position. This sham procedure is different from a control that involves watching a 2D video on a tablet or monitor outside of a headset. Using a true XR sham approach will allow a better understanding of both active and placebo components. It can be helpful to include an intervention arm in which no headset is worn, as this can function as a no-treatment control or a specific factor component control (Table 1). Such a control assesses the influence of XR itself on participant outcomes and can aid in assessing the functioning of the sham. Indeed, a poorly designed sham condition can create more problems than it solves and can fail to solve the problems it seeks to address. As newly developed sham and control conditions become evaluated, validated, and normed, it may become possible to apply them across trials that share features. Additionally, within a trial, it is helpful to monitor patient (and provider) outcome expectations and consider whether the novelty of XR tools is affecting those expectations [28]. Assessing expectations of benefit before starting the trial, perception of benefit during the trial, and requesting that participants guess their trial arm allocation at the end of the trial may conjointly help account for and increase the understanding of expectation effects [28].

As per the definition above, sham approaches involve the viewing of 2D content that contains features similar to MXR content in the active trial arm on a virtual screen within a VR-based room where user orientation is tracked [14,29-31]. At face value, this approach provides reasonable control over many factors involved in MXR delivery, including novelty and immersion, and can account for the look and feel of the content (eg, using nature videos in 2D in parallel to nature-based 360° videos and 3D sounds). As implemented, however, there are several places where sham conditions can fall short, which require further consideration. First, the appropriate sham experience for a given intervention is rarely ready-made. It can be tempting to cobble together an experience that checks boxes for appropriateness; however, we argue that this can backfire and reduce trial quality. In the sham, the 2D video content truly takes center stage and forms the bulk of the experience for the participant in the absence of interactivity and other features. To the extent that this content is uninteresting, poorly curated, or repetitive over time, it has the capacity to result in negative emotions, including frustration and boredom, which are far less likely to be present in active XR interventions [32]. The spare, passive nature of the sham experience is at odds with several tenants of XR design practice [33,34], and extra care may be needed to ensure a positive or neutral experience with the sham over time. Educational XR research has also highlighted the importance of meeting users’ expectations of XR to encourage engagement and effectiveness [35,36]. An XR experience that is disappointing may deflate expectations [33,37] rather than maintaining them like a placebo is meant to do. Therefore, far from serving as a neutral comparison, a sham that violates participants’ expectancies for XR technology capabilities or that makes participants question those capabilities could result in negative responses not typically seen in the active arm, which may be associated with disengagement or drop out [38-41].

## Conclusion

While the question of appropriate controls has been extensively addressed in the clinical trial literature, the use of controls in MXR-focused trials requires careful consideration of unique and relevant XR aspects in combination with other study features. Through the processes of careful design, refining the sham and control conditions, and assessing expectations of improvement and allocation beliefs, we may build a set of evidence-based best practices for moving this field forward. Better controls will enable more robust trials and the development and distribution of more universal knowledge that can be adopted across MXR interventions and their evaluation process. Ultimately, improving our MXR methodology will foster interventions that mitigate suffering, improve clinical outcomes, and optimize treatment performance.

## Abbreviations

MXR: medical extended reality
VR: virtual reality
XR: extended reality
